# Preoperative serum indicators as predictors of postoperative hypoparathyroidism following thyroidectomy

**DOI:** 10.3389/fendo.2025.1594781

**Published:** 2025-09-03

**Authors:** Kailin Liu, Nana Wang, Peng Zhao, Baoyan Zhao, Zhihao Wang, Yongjie Sun, Chong Geng

**Affiliations:** ^1^ Department of Breast and Thyroid Surgery, Shandong Provincial Hospital Affiliated to Shandong First Medical University, Jinan, Shandong, China; ^2^ Second Department of Breast Diseases, Second Department of Thyroid Diseases,Shandong Second Provincial General Hospital No. 4, Jinan, Shandong, China

**Keywords:** postoperative hypoparathyroidism, parathyroid gland, parathyroid hormone, thyroidectomy, predictors

## Abstract

**Background:**

Hypoparathyroidism is a frequent complication following thyroidectomy. However, the risk factors for postoperative hypoparathyroidism remain poorly characterized. This study aims to investigate routine clinical characteristics to identify predictors of postoperative hypoparathyroidism.

**Methods:**

A retrospective analysis was conducted on 3,638 patients who underwent total or hemithyroidectomy with isthmusectomy through open surgery with routine central lymph node dissection. Based on postoperative serum parathyroid hormone(PTH) levels, patients were subdivided according to the normal reference PTH levels or the Q3 quartile of PTH decline rate. Spearman correlation analysis and stepwise multiple linear regression were used to identify factors affecting preoperative serum PTH levels. Univariate and multivariate logistic regression analyses were performed to explore risk factors for postoperative hypoparathyroidism.

**Results:**

Among patients developed postoperative hypoparathyroidism. Multivariate logistic regression revealed that Hashimoto’s thyroiditis (HT) (*OR*=1.786, *P*=0.007) and preoperative blood glucose (*OR*=1.319, *P*=0.005) was an independent risk factor, while body mass index (BMI)≥24 (*OR*=0.714, *P*=0.032),elevated preoperative PTH levels (*OR*=0.988, *P*=0.013)and male (*OR*=0.554, *P*=0.002) were protective factors. Among patients in the Q3 quartile,postoperative PTH decline rate was positively associated with HT (*OR*=2.266, *P<*0.001), preoperative PTH levels (*OR*=1.032, *P<*0.001), and blood glucose (*OR*=1.234, *P*=0.027), while it was negatively associated with BMI≥24 (*OR*=0.611, *P*=0.013), hemoglobin (*OR*=0.984, *P*=0.003), and high-density lipoprotein cholesterol (HDL-C) (*OR*=0.426, *P*=0.007).Additionally, male (*OR*=0.558, *P*=0.003) and preoperative serum Ca^2+^ levels (*OR*=0.110, *P*=0.011) were negatively correlated with the postoperative PTH decline rate in patients undergoing hemithyroidectomy with isthmusectomy. Furthermore, age had no significant effect on the incidence of postoperative hypoparathyroidism.

**Conclusions:**

This study demonstrates that preoperative high glucose levels and HT are risk factors for hypoparathyroidism after total thyroidectomy, while BMI≥24 serves as a protective factor against postoperative hypoparathyroidism. Additionally, female is a risk factor for hypoparathyroidism after both total thyroidectomy and hemithyroidectomy with isthmusectomy, while higher hemoglobin levels and HDL-C are negatively correlated with the decline in PTH levels after total thyroidectomy.

## 1 Introduction

Hypoparathyroidism is a frequent complication following thyroidectomy, characterized by a severe deficiency of parathyroid hormone (PTH). Clinical manifestations of hypoparathyroidism include numbness in the lips and limbs, laryngeal spasm, and respiratory muscle paralysis, all of which significantly impair the quality of life of patients or even are life-threatening. Currently, there is no effective treatment for post-surgical hypoparathyroidism. Despite numerous advancements in surgical techniques, hypoparathyroidism remains a challenge in the field of thyroidectomy (1–3). The incidence of transient hypoparathyroidism ranges from 19% to 38%, and that of permanent hypoparathyroidism varies from 0% to 3% (4). Besides external factors related to surgical procedures such as vascular damage or accidental removal, some studies have indicated that age, sex, and Hashimoto’s thyroiditis are associated with the occurrence of postoperative hypoparathyroidism, suggesting that preoperative status of the parathyroid glands is an important factor (5–7). Some intrinsic factors of the parathyroid glands, such as preoperative parathyroid function, resistance to damage, and recovery ability may also be critical in determining postoperative parathyroid function.

The preoperative status of the parathyroid glands is regulated by various factors. In addition to the direct factors such as 25-hydroxyvitamin D_3_, serum calcium, and phosphorus, recent evidence has shown that waist circumference, BMI, blood glucose (GLU) plasma triglycerides (TG), high-density lipoprotein cholesterol (HDL-C), Homeostatic Model Assessment of Insulin Resistance (HOMA-IR), and leptin levels can also influence parathyroid function (8–11). However, whether these factors also influence the parathyroid glands’ resistance to damage during surgery remains unclear, and existing research remains controversial.

Additionally, due to the high heterogeneity in preoperative PTH levels among patients and the wide range of reference normal PTH levels, some patients suffering significant PTH declines but remain within the reference range, previous studies may overlook these patients with significant PTH declines. Therefore, in this study, we introduced a new threshold method based on the decline rates of PTH levels to investigate the key factors influencing parathyroid function, and identifying the potential risk factors for hypoparathyroidism after thyroidectomy. This study may provide a predictive information for clinical practice, helping the surgeons to perform necessary preventive measures to minimize its incidence.

## 2 Materials and methods

### 2.1 Patients

This retrospective study analyzed the medical records of 3,638 patients with thyroid cancer who underwent (1) Total thyroidectomy (thyroidectomy with bilateral central neck dissection) (2), Hemithyroidectomy with isthmusectomy (lobectomy with isthmusectomy combined with ipsilateral central neck dissection including pretracheal and ipsilateral paratracheal) at the Department of Breast and Thyroid Surgery, Shandong Provincial Hospital, from January 2022 to November 2024. Patients were excluded from this study if they met any of the following criteria: previous history of thyroid or parathyroid surgery, presence of preoperative parathyroid dysfunction, presence of diseases affecting bone and lipid metabolism, or incomplete clinical data. All patients underwent open surgery with routine central lymph node dissection.

### 2.2 Serum PTH level measurement

**Figure 1:**
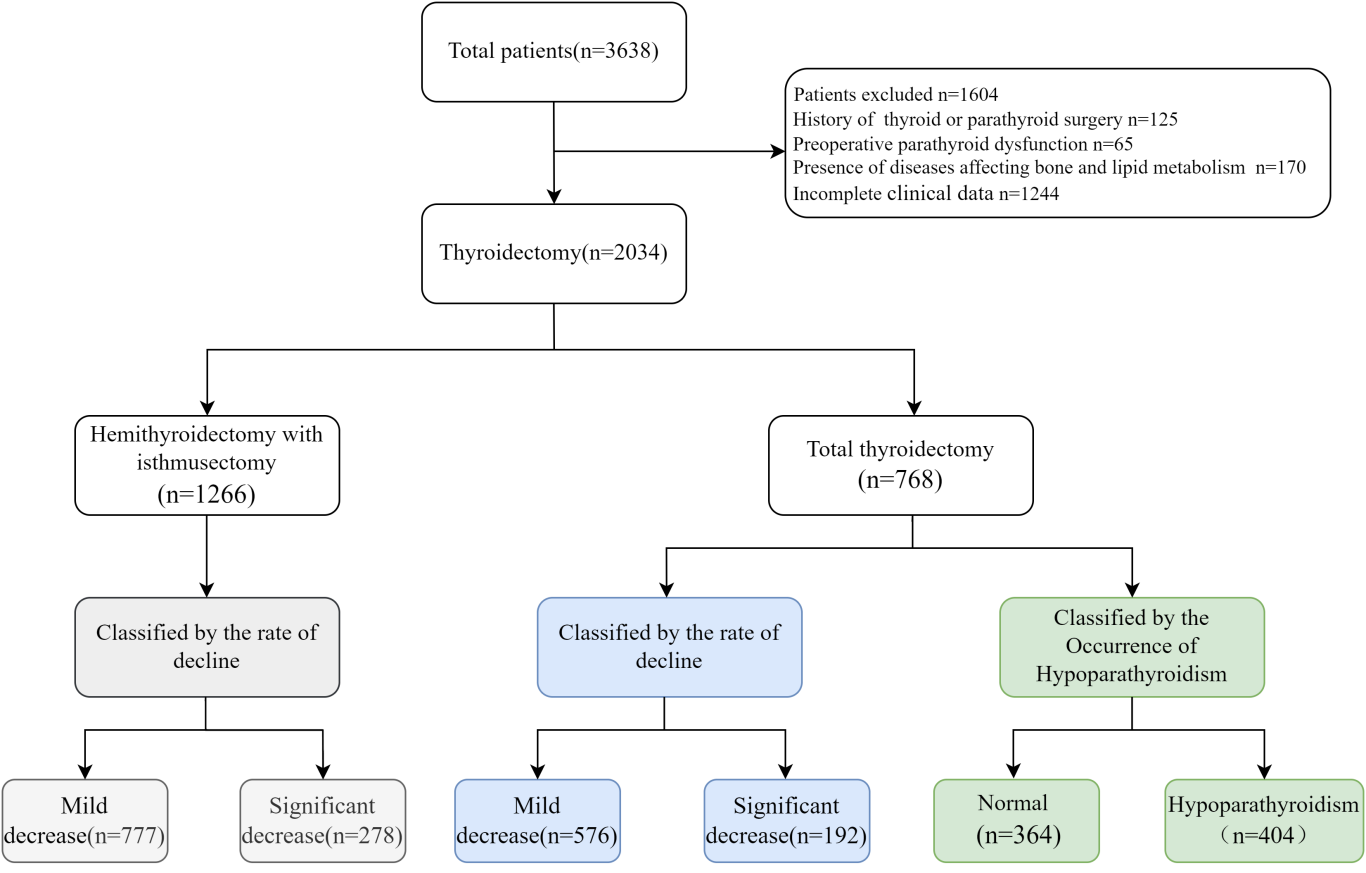
Study flowchart.

Fasting serum samples were collected from patients two days before surgery and one day after surgery. Serum PTH levels were measured using an electrochemiluminescence immunoassay (Roche 801). The normal range for serum PTH was 15–65 pg/mL according to the guidelines. We analyzed the factors influencing postoperative parathyroid function. To avoid potential confounding factors, we first categorized the patients into two groups according to surgery type: total thyroidectomy group and hemithyroidectomy with isthmusectomy (**Figure 1**). In the total thyroidectomy group, 768 eligible patients were included, including 614 females and 154 males, with a mean age of 45.87 ± 11.39 years. We employed two threshold methods to evaluate the decline in postoperative parathyroid functions: (1) the occurrence of postoperative hypoparathyroidism, (2) the Q3 quartile of the rates between postoperative and preoperative PTH levels. Based on the serum PTH levels, patients were divided into two groups: the normal PTH group and the hypoparathyroidism group (serum PTH<15 pg/mL). Additionally, the postoperative PTH decline rate was calculated by (preoperative-postoperative)/preoperative PTH levels, referred to as relative postoperative PTH reduction. Patients were further categorized into two subgroups: significant decline group (>Q3) and mild decline group (<Q3).

### 2.3 Clinical characteristics measurement

Preoperative examinations included serum calcium (Ca²^+^), thyroid function, neck ultrasound, and laryngoscopy. Serum samples were collected two days before and one day after surgery. An automated biochemical analyzer (Beckman AU5800) was used to measure the levels of aspartate transaminase (AST), alanine transaminase (ALT), total bile acid (TBA), total protein (TP), albumin (ALB), triglycerides (TG), total cholesterol (TC), HDL-C, low-density lipoprotein cholesterol (LDL-C), free fatty acids (FFA), blood glucose (GLU), creatinine (CREA), and retinol-binding protein (RBP). Abbott chemiluminescence immunoassay (Alinity i) was used to detect the levels of Free Triiodothyronine(FT3), Free Thyroxine (FT4), and Thyroid-Stimulating Hormone (TSH). Electrochemiluminescence immunoassay (Roche801) was used to detect the levels of TG antibody (TGAb), TPO antibody (TPOAb), and TR antibody (TRAb). Patients with TPOAb≥36 were considered as Hashimoto’s thyroiditis(HT).

### 2.4 Statistical analysis

Statistical analysis was performed using SPSS software(version 27.0). *P*<0.05 (two-sided) was considered statistically significant. The Shapiro-Wilk test was used to assess the normal distribution of data. Normally distributed data were expressed as mean ± SD, and between-group comparisons were performed using independent sample t-tests. Non-normally distributed data were expressed as median (interquartile range), and between-group comparisons were performed using the Mann-Whitney U test. Categorical variables were expressed as frequencies and percentages, and between-group differences were analyzed using Pearson’s *χ*² test with continuity correction. Spearman’s correlation analysis was used to determine variables significantly correlated with serum PTH. Use univariate linear regression analysis to examine the association between clinical examination variables and preoperative serum PTH levels and their changes. Stepwise regression (*P*<0.10) was used to select variables for the multivariate regression model to identify independent influencing factors. Univariate linear regression analysis was performed to examine associations between clinical examination variables and postoperative serum PTH levels and changes. Multivariate logistic regression analysis was performed using the backward likelihood ratio (*LR*) test. Results of multivariate logistic regression were expressed as odds ratios (*OR*s) and 95% confidence intervals (*CI*s). A multivariate predictive model was established, and its predictive performance was evaluated using the receiver operating characteristic (ROC) curve and the area under the curve (AUC).

## 3 Results

### 3.1 Baseline characteristics of the included patients

After reviewing the medical records of a total of 3,638 patients with thyroid cancers in our hospital and 2,034 patients meeting the inclusion criteria as indicated in the method section were analyzed (**Figure 1**). Among them, 1,588 were female, and 446 were male, with an average age of 44.90 ± 11.37 years. The average BMI was 25.13 ± 3.83, preoperative PTH was 46.58 ± 15.77 pg/mL, plasma FFA was 0.41 ± 0.19 mmol/L, TSH was 2.01 ± 1.88 μIU/mL, RBP was 34.46 ± 7.97 mg/L, and Ca²^+^ was 2.33 ± 0.09 mmol/L (**Table 1**).

**Table 1:** Clinical characteristics of patients.

N=2034	Range	Mean (SD)
Age (years)	15.00-78.00	44.90 (11.37)
Height (m)	1.47-1.91	1.64 (0.07)
Weight (kg)	35.00-140.00	68.29 (13.28)
BMI (kg/m^2^)	14.76-43.21	25.13 (3.83)
Erythrocyte (10^12/L)	2.51-5.85	4.41 (0.43)
Hemoglobin (g/L)	68.00-176.00	129.35 (16.32)
FT3 (pmol/L)	1.77-9.03	4.26 (0.5)
FT4 (pmol/L)	1.06-44.44	12.35 (1.79)
TSH (μIU/mL)	0.01- 49.61	2.01 (1.88)
TGAb (IU/mL)	10.00 -4000.00	90.21 (320.44)
TPOAb (IU/mL)	9.00-600.00	38.25 (96.02)
TRAb (IU/L)	0.80-40.00	0.89 (1.12)
AST (U/L)	2.00-725.00	20.94 (17.57)
ALT (U/L)	1.00-755.00	20.26 (22.68)
TBA (μmol/L)	0.30-72.40	5.08 (4.41)
TP (g/L)	52.60-87.00	68.79 (4.72)
ALB (g/L)	30.20-97.30	41.26 (3.34)
TG (mmol/L)	0.33-44.58	1.42 (1.41)
TC (mmol/L)	1.93-52.90	4.76 (1.44)
HDL_C (mmol/L)	0.45-2.66	1.32 (0.31)
LDL_C (mmol/L)	0.71-6.45	2.95 (0.75)
FFA (mmol/L)	0.07 -1.46	0.41 (0.19)
GLU (mmol/L)	3.02-13.98	4.67 (0.87)
CREA (μmol/L)	32.10-163.50	57.21 (11.71)
RBP (mg/L)	17.60-73.50	34.46 (7.97)
Pre_Ca^2+^ (mmol/L)	2.09-2.78	2.33 (0.09)
Pre_PTH (pg/mL)	14.41-124.60	46.58 (15.77)

Preoperative Ca^2+^ (Pre_Ca^2+^), Preoperative PTH (Pre_PTH).

### 3.2 Factors influencing preoperative parathyroid functions

**Figure 2:**
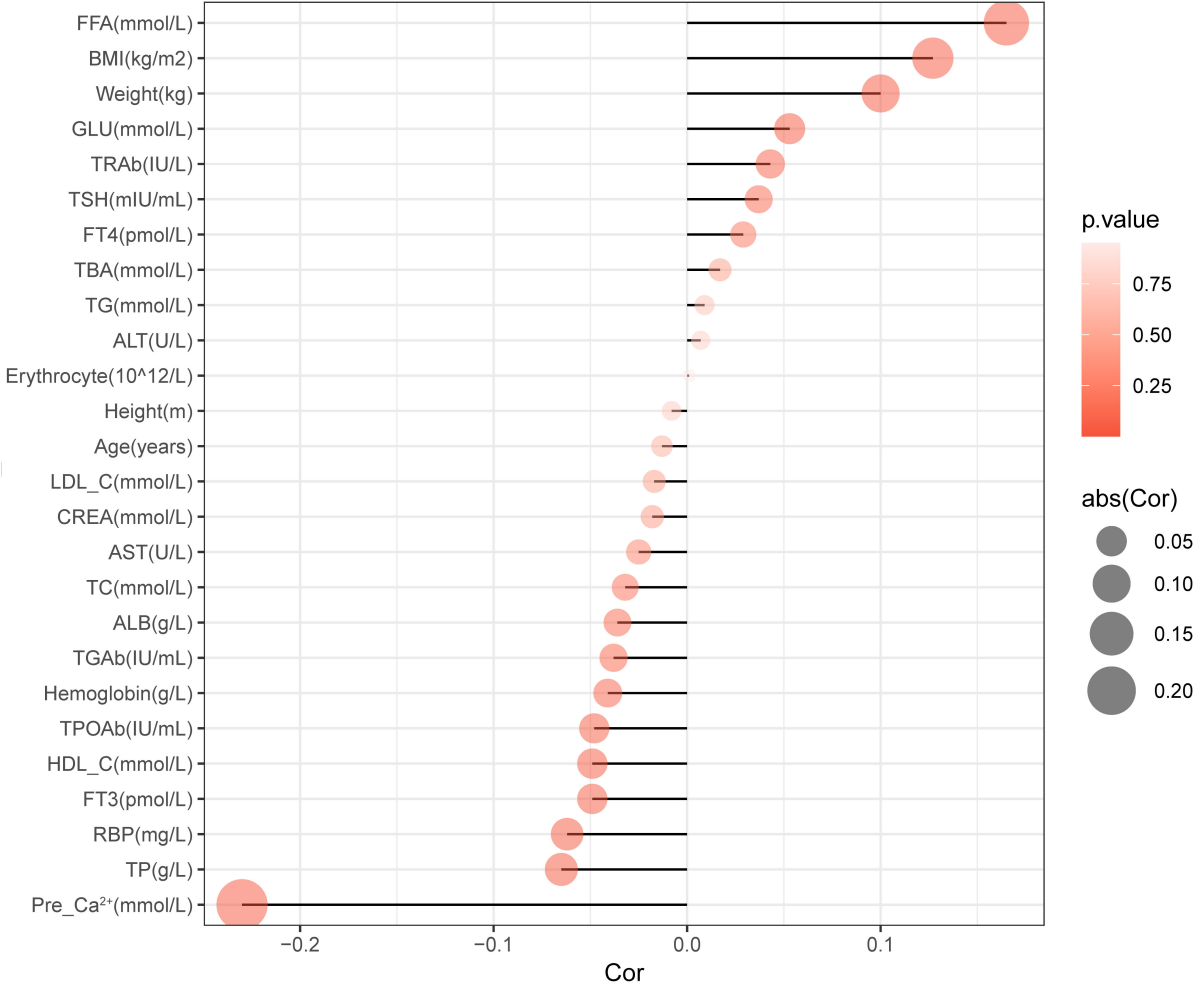
Correlation chart of preoperative PTH influencing factors.

We firstly analyzed the factors which may influence the preoperative parathyroid functions. Spearman correlation analysis was used to identify variables significantly associated with preoperative serum PTH levels, as shown in **Table 2**. We found that the preoperative serum PTH levels were positively correlated with BMI (*R*=0.127, *P<*0.001), Weight (*R*=0.1, *P<*0.001), FFA (*R*=0.165, *P<*0.001), and GLU (*R*=0.053, *P=*0.016); and negatively correlated with FT3 (*R*=-0.049, *P*=0.027), TPOAb (*R*=-0.048, *P*=0.03), TP (*R*=-0.065, *P*=0.004), HDL-C (*R*=-0.049, *P*=0.026), and RBP (*R*=-0.062, *P*=0.005) (**Figure 2**).

**Table 2:** Preoperative PTH spearman correlation analysis.

N=2034	*R*	*P*
Age (years)	-0.013	0.544
Height (m)	-0.008	0.714
Weight (kg)	0.100^**^	<0.001
BMI (kg/m^2^)	0.127^**^	<0.001
Erythrocyte (10^12/L)	0.001	0.950
Hemoglobin (g/L)	-0.041	0.063
FT3 (pmol/L)	-0.049^*^	0.027
FT4 (pmol/L)	0.029	0.187
TSH (μIU/mL)	0.037	0.097
TGAb (IU/mL)	-0.038	0.084
TPOAb (IU/mL)	-0.048^*^	0.030
TRAb (IU/L)	0.043	0.052
AST (U/L)	-0.025	0.267
ALT (U/L)	0.007	0.765
TBA (μmol/L)	0.017	0.446
TP (g/L)	-0.065^**^	0.004
ALB (g/L)	-0.036	0.103
TG (mmol/L)	0.009	0.681
TC (mmol/L)	-0.032	0.149
HDL_C (mmol/L)	-0.049^*^	0.026
LDL_C (mmol/L)	-0.017	0.439
FFA (mmol/L)	0.165^**^	<0.001
GLU (mmol/L)	0.053^*^	0.016
CREA (μmol/L)	-0.018	0.408
RBP (mg/L)	-0.062^**^	0.005
Pre_Ca^2+^ (mmol/L)	-0.230^**^	<0.001

Preoperative Ca^2+^ (Pre_Ca^2+^).

*P < 0.05. **P < 0.01.

Based on comprehensive stepwise regression analysis and clinical practice, this study included the following 12 factors revealed from univariate regression analysis (**Supplementary Table S1**) for multiple linear regression analysis. The results showed that preoperative serum PTH levels were positively correlated with FFA (*β*=0.177, *P*<0.001), BMI (*β*=0.152, *P*<0.001), TSH (*β*=0.059, *P*=0.006), and FT4 (*β*=0.047, *P*=0.033), while negatively correlated with RBP (*β*=-0.049, *P*=0.036) and Ca²^+^ (*β*=-0.235, *P*<0.001) (**Table 3**).

**Table 3:** Multiple stepwise regression analysis of preoperative PTH influencing factors.

Variables	SE	*β*	t	P
Constant	9.262		12.667	<0.001
Pre_Ca^2+^ (mmol/L)	4.509	-0.263	-10.229	<0.001
FFA (mmol/L)	1.856	0.171	7.746	<0.001
BMI (kg/m^2^)	0.092	0.153	6.872	<0.001
TSH (μIU/mL)	0.18	0.057	2.654	0.008
ALB (g/L)	0.126	0.063	2.503	0.012
RBP (mg/L)	0.047	-0.058	-2.434	0.015
FT4 (pmol/L)	0.192	0.046	2.093	0.036

*β*: Standardized Coefficient, Preoperative Ca^2+^(Pre_Ca^2+^).

### 3.3 Factors influencing postoperative parathyroid functions

#### 3.3.1 Total thyroidectomy subgroup

The difference in postoperative clinical characteristics between normal and hypoparathyroidism patients are shown in **Table 4**. Using the first method, based on the occurrence of postoperative hypoparathyroidism, 364 patients (47.4%) remained within the normal postoperative serum PTH range, while 404 (52.6%) developed hypoparathyroidism. Considering the high heterogeneity in preoperative PTH levels among individuals and wide range of reference PTH levels, relying solely on reference PTH levels to identify the risks for postoperative hypoparathyroidism may overlook patients with significant PTH declines but remain within the reference range. Therefore, we employed two threshold methods to evaluate the decline in postoperative parathyroid function as described in the methods section.

**Table 4:** Comparison of baseline characteristics in total thyroidectomy patients.

Variables	Classified by the occurrence of hypoparathyroidism		χ^2^/t/Z	P	Classified by the rate of decline		χ^2^/t/Z	P
	Normal (n=364)	Hypoparathyroidism (n=404)			Mild decrease (n=576)	Significant decrease (n=192)		
Sex,*n* (%)			11.773	<0.001			3.13	0.077
female	272 (74.7)	342 (84.7)			452 (78.5)	162 (84.4)		
male	92 (25.3)	62 (15.3)			124 (21.5)	30 (15.6)		
Age,*n* (%)			0.434	0.51			0.506	0.477
<45	169 (46.4)	178 (44.1)			256 (44.4)	91 (47.4)		
≥45	195 (53.6)	226 (55.9)			320 (55.6)	101 (52.6)		
BMI,*n* (%)			5.456	0.019			2.332	0.127
<24	132 (36.3)	180 (44.6)			225 (39.1)	87 (45.3)		
≥24	232 (63.7)	224 (55.4)			351 (60.9)	105 (54.7)		
TPOAb,*n* (%)			8.316	0.004			14.145	<0.001
<36	325 (89.3)	331 (81.9)			510 (88.5)	149 (77.6)		
≥36	39 (10.7)	73 (18.1)			66 (11.5)	43 (22.4)		
Erythrocyte (10^12/L)	4.43 (4.18,4.72)	4.38 (4.13,4.63)	-2.11	0.035	4.39 (4.16,4.68)	4.39 (4.14,4.68)	-0.563	0.573
Hemoglobin (g/L)	131 (123, 142)	128 (119.75, 137)	3.09	0.002	131 (122,140)	127 (117.5,135.25)	-3.206	0.001
FT3 (pmol/L)	4.23 (3.95, 4.56)	4.2 (3.92, 4.49)	1.30	0.194	4.22 (3.94,4.53)	4.18 (3.93,4.51)	-0.834	0.404
FT4 (pmol/L)	12.21 (11.22, 13.23)	12.2 (11.19, 13.21)	0.01	0.992	12.14 (11.15,13.2)	12.36 (11.44,13.34)	-1.856	0.063
TSH (μIU/mL)	1.67 (1.07,2.39)	1.7 (1.16,2.44)	-0.894	0.371	1.69 (1.16,2.43)	1.66 (1.08,2.3)	-0.724	0.469
TGAb (IU/mL)	16.5 (14,36.78)	17.1 (14.5,67.8)	-2.10	0.036	16.6 (14.2,47.72)	17.15 (14.47,69.82)	-1.203	0.229
TRAb (IU/L)	0.8 (0.8,0.8)	0.8 (0.8,0.8)	-0.99	0.321	0.8 (0.8,0.8)	0.8 (0.8,0.8)	-0.34	0.734
AST (U/L)	20 (16.75,23)	19 (16,22)	-1.58	0.114	19 (16,23)	19 (16,22)	-1.355	0.176
ALT (U/L)	16 (12,24)	15 (11,22)	-1.25	0.211	15 (12,23)	15 (11,22)	-0.763	0.446
TBA (μmol/L)	3.9 (2.5,6.23)	3.95 (2.8,6.2)	-0.47	0.64	3.9 (2.5,6.23)	4 (2.88,6.12)	-0.661	0.509
TP (g/L)	69.4±4.84	68.93±4.76	0.89	0.375	69.25±4.86	68.56±4.59	-1.512	0.13
ALB (g/L)	41.3 (39.27, 43.23)	40.7 (38.98, 42.7)	1.72	0.086	40.95 (39.2,43)	40.8 (39,42.8)	-0.827	0.409
TG (mmol/L)	1.23 (0.86,1.69)	1.15 (0.81,1.65)	-1.05	0.293	1.2 (0.85,1.65)	1.19 (0.8,1.71)	-0.003	0.998
TC (mmol/L)	4.7 (4.1,5.27)	4.71 (4.05,5.5)	-0.05	0.958	4.71 (4.1,5.37)	4.63 (3.99,5.48)	-0.601	0.548
HDL_C (mmol/L)	1.28 (1.06, 1.5)	1.31 (1.14, 1.5)	-1.07	0.287	1.32 (1.1,1.52)	1.25 (1.11,1.44)	-1.753	0.08
LDL_C (mmol/L)	2.95 (2.44,3.44)	2.94 (2.4,3.46)	-0.22	0.829	2.95 (2.45,3.44)	2.9 (2.37,3.57)	-0.407	0.684
FFA (mmol/L)	0.37 (0.26,0.51)	0.38 (0.27,0.51)	-0.50	0.62	0.37 (0.27,0.51)	0.39 (0.29,0.52)	-1.035	0.301
GLU (mmol/L)	4.53 (4.2, 4.91)	4.52 (4.16, 4.92)	-2.02	0.039	4.53 (4.18,4.91)	4.52 (4.19,4.93)	-0.408	0.683
CREA (μmol/L)	56.15 (49.27,64.23)	53.9 (48.8, 61)	2.05	0.04	55.5 (49.4,63.23)	53.45 (47.68,60)	-2.445	0.014
RBP (mg/L)	33.5 (29.3, 38.92)	32.2 (28.85, 38.12)	2.01	0.045	33.1 (29.1,38.52)	32.05 (28.9,37.92)	-1.116	0.264
Pre_Ca^2+^ (mmol/L)	2.34 (2.28, 2.39)	2.33 (2.27, 2.38)	0.72	0.472	2.34 (2.28,2.39)	2.32 (2.26,2.38)	-2.35	0.019
Pre_PTH (pg/mL)	46.04 (37.06,56.8)	43.44 (34.25,55.52)	-2.41	0.016	42.94 (34.62,52.34)	52.08 (40.58,61.74)	-6.197	<0.001

Data are expressed as means ± SD or medians (interquantile range), Preoperative Ca^2+^ (Pre_Ca^2+^) Preoperative PTH (Pre_PTH).

**Figure 3:**
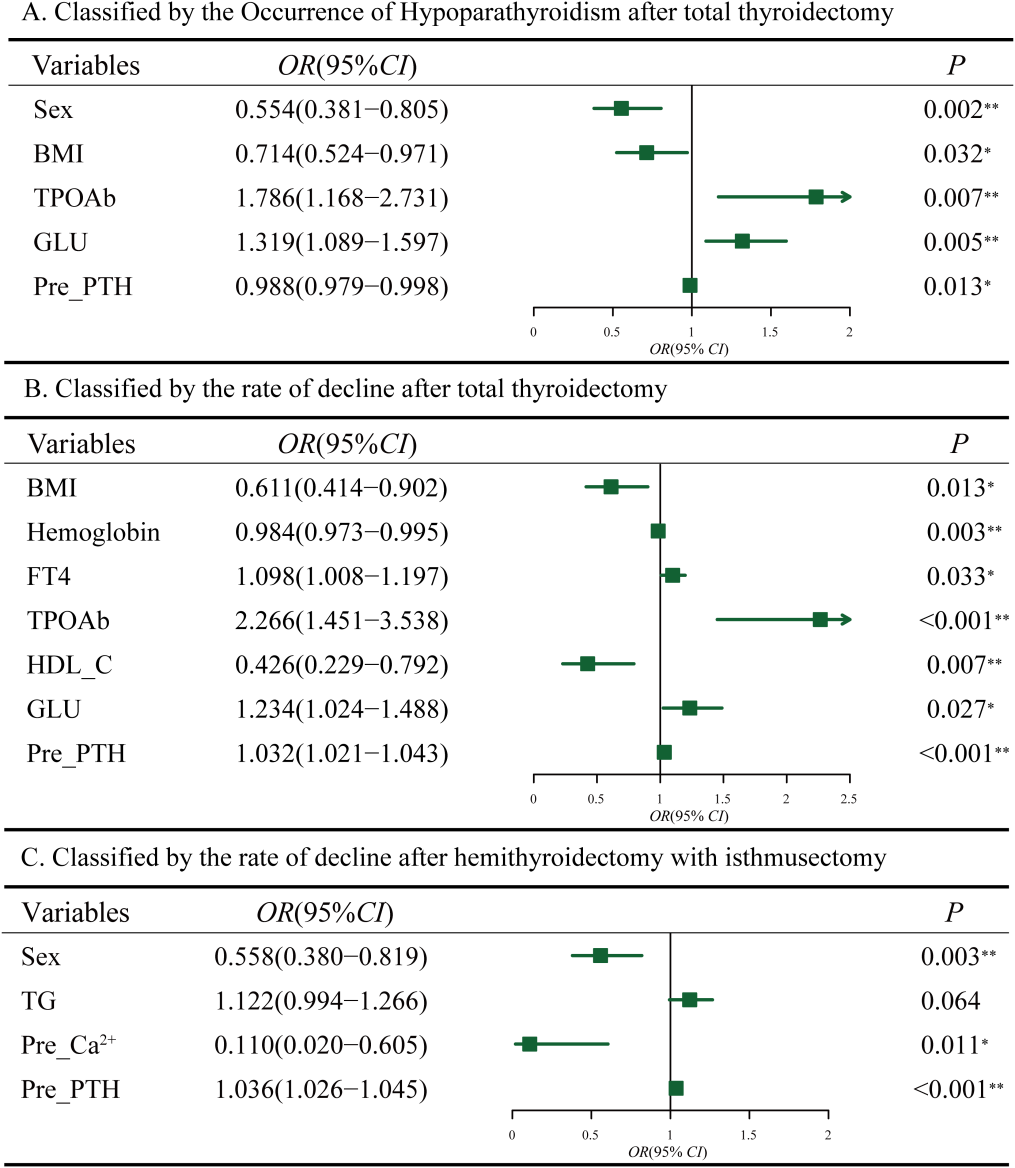
Multifactorial analysis of hypoparathyroidism after thyroidectomy. **(A)** Classified by the occurrence of hypoparathyroidism after total thyroidectomy. **(B)** Classified by the rate of postoperative PTH decline after total thyroidectomy. **(C)** Classified by the rate of postoperative PTH decline after hemithyroidectomy with isthmusectomy. Preoperative Ca^2+^ (Pre_Ca^2+^), Preoperative PTH (Pre_PTH).

Using the first method, univariate logistic regression analysis revealed 8 variables significantly associated with the occurrence of hypoparathyroidism (*P*<0.05, **Supplementary Table S2**, left). Multivariate logistic regression analysis was conducted on variables with *P*<0.1 in univariate analysis to identify independent risk factors for postoperative hypoparathyroidism. As shown in (**Figure 3A**), HT (*OR*=1.786, 95% *CI*: 1.168-2.731, *P*=0.007), and GLU (*OR*=1.319, 95% *CI*:1.089-1.597, *P*=0.005) were identified as independent risk factors for postoperative hypoparathyroidism, while BMI≥24 (*OR*=0.714, 95% *CI*: 0.524-0.971, *P*=0.032), elevated preoperative PTH levels (*OR*=0.988, 95% *CI*: 0.979-0.998, *P*=0.013) and male (*OR*=0.554, 95% *CI*: 0.381-0.805, *P*=0.002) were protective factors.

Using the second method, based on the Q3 quartile of the rates between postoperative and preoperative PTH levels, a total of 576 patients (75%) were classified into the mild decrease group (<Q3), while 192 patients (25%) were classified into the significant decrease group (>Q3) (**Table 4**). Univariate logistic regression analysis revealed 6 variables that were significantly associated with the significant decline in PTH levels after total thyroidectomy. Multivariate logistic regression analysis on variables with *P*<0.1 in univariate analysis (**Supplementary Table S2**, right) revealed that FT4 (*OR*=1.098, 95% *CI*:1.008-1.197, *P*=0.033), HT (*OR*=2.266, 95% *CI*: 1.451-3.538, *P<*0.001) and GLU (*OR*=1.234, 95% *CI*:1.024-1.488, *P*=0.027) were independent risk factors for significant postoperative PTH decline. BMI≥24 (*OR*=0.611, 95% *CI*: 0.414-0.902, *P*=0.013), Hemoglobin (*OR*=0.984, 95% *CI*: 0.973-0.995, *P=*0.003) and HDL-C (*OR*=0.426, 95% *CI*: 0.229-0.792, *P*=0.007) were identified as protective factors (**Figure 3B**). The area under the ROC curve (AUC) of the multivariable combined model was 0.698 (95% *CI*: 0.655–0.742), while that of preoperative PTH alone was 0.649 (95% *CI*:0.605-0.694) (**Supplementary Figure S1A**).

#### 3.3.2 Hemithyroidectomy with isthmusectomy subgroup

Among 1,055 patients undergoing hemithyroidectomy with isthmusectomy, only 69 (6.44%) of these patients showed postoperative PTH levels below the normal reference range. Therefore, we focused on the second method, which resulted in 777 patients (75%) in the mild decline group and 278 (25%) in the significant decline group (**Table 5**). Multivariate logistic regression analysis on variables with *P <*0.1 in univariate analysis (**Supplementary Table S3**) indicated that male (*OR=*0.558, 95% *CI:* 0.380-0.819*, P=*0.003) and serum Ca²^+^ levels (*OR=*0.110, 95% *CI:* 0.020-0.605*, P=*0.011) were negatively associated with postoperative PTH decline, while preoperative serum PTH levels (*OR*=1.036, 95% *CI*: 1.026-1.045, *P*<0.001) were positively associated (**Figure 3C**). The area under the ROC curve (AUC) of the multivariable combined model was 0.668 (95% *CI*: 0.631-0.706), while that of preoperative PTH alone was 0.683 (95% *CI*: 0.646-0.720) (**Supplementary Figure S1B**).Given that hemithyroidectomy does not affect the contralateral glands, these preoperative indicators may be also associated with an insufficient compensatory capacity of the contralateral gland(s).

**Table 5:** Comparison of baseline characteristics between mild and significant postoperative PTH decline in hemithyroidectomy with isthmusectomy patients.

Variables	Mild decrease (n=777)	Significant decrease (n=278)	χ^2^/*Z*	*p*
Sex,*n* (%)			12.187	<0.001
female	577 (74)	235 (85)		
male	200 (26)	43 (15)		
Age,*n* (%)			0.935	0.334
<45	393 (51)	150 (54)		
≥45	384 (49)	128 (46)		
BMI,*n* (%)			0.122	0.726
<24	328 (42)	114 (41)		
≥24	449 (58)	164 (59)		
TPOAb,*n* (%)				
<36	667 (85.8)	238 (85.6)	0.003	0.524
≥36	110 (14.2)	40 (14.4)		
Erythrocyte (10^12^/L)	4.39 (4.13,4.7)	4.33 (4.06,4.56)	-2.925	0.003
Hemoglobin (g/L)	130 (121,141)	128 (119,136)	-2.889	0.004
FT3 (pmol/L)	4.24 (3.99,4.55)	4.17 (3.92,4.46)	-2.939	0.003
FT4 (pmol/L)	12.24 (11.33,13.11)	12.06 (11.32,13.2)	-0.471	0.638
TSH (μIU/mL)	1.68 (1.21,2.45)	1.85 (1.32,2.64)	-2.139	0.032
TGAb (IU/mL)	16.2 (14.1,25.6)	16.5 (14,41.4)	-0.022	0.983
TRAb (IU/L)	0.8 (0.8,0.8)	0.8 (0.8,0.8)	-1.295	0.195
AST (U/L)	19 (16,22)	18 (16,21)	-1.957	0.05
ALT (U/L)	15 (11,23)	15 (11,19.75)	-1.289	0.198
TBA (μmol/L)	3.9 (2.5,6.4)	3.9 (2.5,6.2)	-0.256	0.798
TP (g/L)	68.4 (65.4,71.8)	68.05 (65.43,71.47)	-0.322	0.748
ALB (g/L)	41.2 (39.5,43)	40.8 (38.92,42.6)	-1.952	0.051
TG (mmol/L)	1.16 (0.83,1.74)	1.08 (0.8,1.7)	-0.742	0.458
TC (mmol/L)	4.65 (4.06,5.31)	4.63 (4.09,5.2)	-0.242	0.809
HDL_C (mmol/L)	1.29 (1.09,1.54)	1.29 (1.14,1.47)	-0.218	0.827
LDL_C (mmol/L)	2.86 (2.4,3.38)	2.89 (2.43,3.39)	-0.011	0.991
FFA (mmol/L)	0.38 (0.28,0.52)	0.41 (0.29,0.54)	-1.67	0.095
GLU (mmol/L)	4.53 (4.2,4.93)	4.56 (4.26,4.89)	-0.635	0.525
CREA (μmol/L)	55.7 (49.6,63.6)	54.85 (49.5,61.03)	-1.641	0.101
RBP (mg/L)	33.5 (29.1,38.9)	32.75 (28,37.75)	-2.072	0.038
Pre_Ca^2+^ (mmol/L)	2.33 (2.28,2.39)	2.3 (2.25,2.36)	-4.283	<0.001
Pre_PTH (pg/mL)	41.39 (34.38,52.84)	53.22 (40.02,64.41)	-8.341	<0.001

Preoperative Ca^2+^ (Pre_Ca^2+^), Preoperative PTH (Pre_PTH).

## 4 Discussion

Hypoparathyroidism is a frequent complication following thyroidectomy (12). In this study, we employed two methods to define hypoparathyroidism and to identify preoperative risk factors for postoperative hypoparathyroidism in patients undergoing total thyroidectomy or hemithyroidectomy with isthmusectomy. Although surgical technique is considered a direct factor influencing postoperative hypoparathyroidism, our study still revealed significant associations between this complication and certain specific clinical indicators.

In the total thyroidectomy group, we found that preoperative high glucose levels are a risk factor for postoperative hypoparathyroidism, while high BMI is a protective factor for postoperative hypoparathyroidism in both methods to define the decline of parathyroid function. Additionally, preoperative high PTH levels were identified as an independent risk factor for a significant postoperative PTH decline. Although we identified preoperative high glucose levels and elevated preoperative PTH levels as independent risk factors, these two may be interrelated, as preoperative serum PTH levels have been reported to positively correlate with blood glucose levels (13–15). The elevated glucose levels seem to be a consequence of higher PTH levels as increased serum PTH levels may impair insulin signaling by inhibiting IRS-1 serine phosphorylation in adipocytes and reducing whole-body fat oxidation (15). However, we found that blood glucose levels affected both postoperative absolute PTH values and relative postoperative PTH reduction. Following thyroidectomy, most patients experience a varying degree of PTH decline. We observed that preoperative high glucose levels are a risk factor for both postoperative hypoparathyroidism and significant PTH reduction. This finding further highlights the complex and intricate interplay among blood glucose, PTH, and insulin, holding important implications for predicting the risk of postoperative hypoparathyroidism. Nevertheless, the mechanisms remain unclear and warrant further investigation in future studies.

The protective role of higher BMI is consistent with previous studies (16–19), though studies involving more than 2,000 patients remain relatively rare. Finel et al. found that although patients with BMI≥25 experience significantly longer surgical durations, the incidence of transient or permanent hypoparathyroidism does not show statistical significance (19). Based on statistical analysis of over 2,000 patients, our study identified BMI≥24 as a protective factor against hypoparathyroidism. Increased adipose tissue within the anatomical region, particularly within the parathyroid capsule, may contribute to improved blood supply to the parathyroid glands during surgery, providing a protective effect for patients with higher BMI (17). However, we believe the more crucial factor is that the increased adipose tissue surrounding the parathyroid glands acts as a buffer, reducing physical trauma to the glands and their blood vessels during surgical manipulation. This buffering effect may further lower the risk of parathyroid dysfunction.

To date, no studies have elucidated the effect of Retinol-Binding Protein(RBP) on PTH levels. Our data show a negative correlation between preoperative serum PTH levels and RBP, and univariate analysis identified high RBP levels as a risk factor for postoperative hypoparathyroidism. RBP is currently the only known specific transport protein for vitamin A (retinol). After retinol binds to RBP, it further forms a retinol-RBP-TTR complex with transthyretin (TTR), which transports retinol to target tissues (20). Retinoic acid (RA), the active metabolite of retinol, plays a crucial role in regulating cell proliferation and differentiation. Previous studies have confirmed that parathyroid cells are among the few specialized cell types equipped with a complete vitamin A metabolic pathway, expressing key proteins essential for intracellular retinol and retinoic acid metabolism. These include 9-cis-retinoic acid receptor (RXR), all-trans-retinoic acid receptor (RAR), cellular retinol-binding protein (CRBP type I), and cellular retinoic acid-binding proteins (CRABP types I and II) (21, 22).

Retinoic acid can inhibit the proliferation of parathyroid chief cells and significantly reduce PTH mRNA expression levels by activating nuclear receptor signaling pathways, thereby suppressing PTH secretion. Additionally, retinoic acid enhances the calcium sensitivity of parathyroid cells by shifting the calcium set point for PTH release to the left, allowing PTH secretion to be suppressed at lower blood calcium levels. The observed negative correlation between RBP levels and PTH levels in our study is likely due to RBP interfering with retinol metabolism (21, 23). Although these findings suggest that RBP may play a role in regulating parathyroid cell function, the exact mechanisms remain unclear and warrant further investigation.

Although Hashimoto’s thyroiditis (HT) is widely recognized as a key risk factor for postoperative hypoparathyroidism and a predictor of incidental parathyroidectomy (24, 25), some studies have suggested no association between HT and postoperative hypoparathyroidism in patients undergoing total thyroidectomy (26, 27). Our study further demonstrated a positive correlation between postoperative serum PTH decline and TPOAb levels — a crucial marker for HT diagnosis aside from histological evidence — indicating a close association between HT and postoperative serum PTH reduction. This finding aligns with other studies that have identified HT as an independent risk factor for hypoparathyroidism (5, 28, 29). Moreover, research has shown that TPOAb levels are closely correlated with the number of autoreactive lymphocytes infiltrating the thyroid gland. Higher TPOAb levels are associated with more severe thyroid destruction and fibrosis (30). We propose that long-term HT may lead to thyroid enlargement, resulting in a grayish appearance of the gland, hard in texture, and exert chronic compression on the parathyroid glands, potentially resulting in structural and functional alterations. Additionally, during surgery on HT patients, thyroid enlargement, increased hardness, and adhesions to surrounding tissues elevate the difficulty of dissection (31, 32). The increased vascularity of the thyroid can also cause intraoperative bleeding and obscure the surgical field (33, 34), further raising the risk of parathyroid injury. On the other hand, the heightened risk of parathyroid injury in HT patients may be linked to the intense inflammatory process. The dense fibrous capsule formed by inflammation may obscure parathyroid glands located within or near the capsule, making them harder to identify and increasing the likelihood of accidental resection (5, 28, 35).

Currently, whether sex factors influence hypoparathyroidism after thyroidectomy remains controversial (5, 36–38). Another key finding of our study is that sex is a significant factor influencing postoperative hypoparathyroidism, regardless of whether patients underwent total thyroidectomy or hemithyroidectomy with isthmusectomy. This finding is consistent with the systematic review by Edafe et al (4). However, the underlying mechanisms remain unclear. Anatomical and physiological studies suggest potential differences between male and female parathyroid glands. For example, autopsy studies have shown that male parathyroid glands may contain a higher proportion of stromal fat than those in females (39). Additionally, sex-related differences may exist in factors regulating monoclonal proliferation and mitosis in parathyroid tissue (37, 40).

Hormonal differences are another possible explanation. For instance, estrogen has been shown to regulate intestinal calcium absorption independently of 1,25(OH)_2_D (41–43), contributing to the maintenance of serum Ca²^+^ levels. *In vitro* studies have confirmed the presence of estrogen receptors in the parathyroid glands, revealing a functional relationship between estrogen and the parathyroid, which can directly influence PTH secretion, DNA synthesis, and gene transcription (44–46). Meanwhile, the prevalence of HT in females is significantly higher than in males, with the male-to-female ratio being approximately 1:10 (47). As discussed above, HT has been established as an independent risk factor for parathyroid injury, which may be a key contributor to the observed sex-differences. The interplay of these factors may explain why female patients are more likely to experience postoperative serum PTH decline and related complications after thyroidectomy. It is worth noting that in this study, the BMI of male patients was significantly higher than that of female patients, suggesting that the protective effect observed in males may be partly attributable to their higher BMI.

Previous studies on the effect of age on postoperative hypoparathyroidism have yielded conflicting conclusions, which can be broadly categorized into three groups. Some studies suggest that older patients are more prone to developing postoperative hypoparathyroidism (6, 48), while others report that the complication is more common among young patients (26, 49, 50). Additionally, some studies have found no significant association between age and postoperative hypoparathyroidism (36, 51). Our study found that age had no significant impact on postoperative hypoparathyroidism or relative postoperative PTH reduction. This finding suggests that clinicians should focus more on other potential risk factors rather than relying solely on patient age to evaluate the risk of postoperative parathyroid dysfunction.

Lastly, we found a negative correlation between preoperative high hemoglobin levels and postoperative PTH decline. This may be because relatively higher hemoglobin levels help protect the parathyroid glands from ischemic and hypoxic injury during surgery. Moreover, hemoglobin may further reduce the risk of postoperative PTH decline by regulating microcirculation and improving local tissue metabolism (52). Therefore, assessing preoperative hemoglobin levels could serve as a potential indicator for predicting postoperative hypoparathyroidism, providing a new reference for perioperative management.

The strength of this study lies in its large sample size and comprehensive analysis, providing robust data support for exploring the relationship between preoperative indicators and postoperative hypoparathyroidism. Also, we developed a new threshold method to better evaluate the decline in postoperative parathyroid function. However, there are still certain limitations in this study. The retrospective single-center design may affect the generalizability of the results. Moreover, this study did not consider the potential impact of magnesium levels on parathyroid function. Future studies should incorporate data from multiple centers, include a more diverse population, and assess additional relevant indicators to enhance the representativeness and applicability of the findings.

## 5 Conclusion

Our study indicates that preoperative serum PTH levels are positively correlated with FFA, BMI, TSH, and FT4, while negatively correlated with RBP and Ca^2+^. Higher preoperative blood glucose levels and HT are risk factors for hypoparathyroidism after total thyroidectomy, whereas BMI≥24 is a protective factor for postoperative hypoparathyroidism in both the two methods to define the decline of parathyroid function. Additionally, female is a risk factor for hypoparathyroidism after total or hemithyroidectomy with isthmusectomy. Higher preoperative hemoglobin levels and HDL-C are negatively correlated with the decline of PTH after total thyroidectomy.

## Data Availability

The raw data supporting the conclusions of this article will be made available by the authors, without undue reservation.
