# Supplementary material for: Preoperative serum indicators as predictors of postoperative hypoparathyroidism following thyroidectomy
Source: Front Endocrinol (Lausanne). 2025 Sep 3;16:1594781. doi: 10.3389/fendo.2025.1594781 (PMC12440752; doi:10.3389/fendo.2025.1594781)
Supplement: Supplementary file 1 [file DataSheet1.docx]

**(TableS1) Univariate linear regression of preoperative PTH**

| Variables | *β* | SE | t | p |
| --- | --- | --- | --- | --- |
| Age(years) | -0.004 | 0.031 | -0.167 | 0.868 |
| Height(m) | -0.022 | 4.782 | -1.002 | 0.316 |
| Weight(kg) | 0.105 | 0.026 | 4.746 | <0.001 |
| BMI(kg/m2) | 0.149 | 0.090 | 6.796 | <0.001 |
| Erythrocyte(10^12/L) | 0.005 | 0.814 | 0.218 | 0.828 |
| Hemoglobin(g/L) | -0.046 | 0.021 | -2.076 | 0.038 |
| FT3(pmol/L) | -0.041 | 0.698 | -1.851 | 0.064 |
| FT4(pmol/L) | 0.021 | 0.195 | 0.969 | 0.333 |
| TSH(μIU/mL) | 0.046 | 0.186 | 2.063 | 0.039 |
| TGAb(IU/mL) | -0.036 | 0.001 | -1.643 | 0.101 |
| TPOAb(IU/mL) | -0.014 | 0.004 | -0.625 | 0.532 |
| TRAb(IU/L) | 0.025 | 0.313 | 1.146 | 0.252 |
| AST(U/L) | -0.008 | 0.020 | -0.378 | 0.706 |
| ALT(U/L) | 0.007 | 0.015 | 0.327 | 0.743 |
| TBA(μmol/L) | -0.004 | 0.079 | -0.190 | 0.849 |
| TP(g/L) | -0.062 | 0.074 | -2.791 | 0.005 |
| ALB(g/L) | -0.039 | 0.105 | -1.770 | 0.077 |
| TG(mmol/L) | -0.001 | 0.248 | -0.025 | 0.980 |
| TC(mmol/L) | -0.005 | 0.242 | -0.245 | 0.806 |
| HDL_C(mmol/L) | -0.061 | 1.133 | -2.747 | 0.006 |
| LDL_C(mmol/L) | -0.015 | 0.465 | -0.680 | 0.496 |
| FFA(mmol/L) | 0.151 | 1.839 | 6.892 | <0.001 |
| GLU(mmol/L) | 0.045 | 0.403 | 2.036 | 0.042 |
| CREA(μmol/L) | -0.023 | 0.030 | -1.015 | 0.310 |
| RBP(mg/L) | -0.062 | 0.044 | -2.799 | 0.005 |
| Pre_Ca^2+^(mmol/L) | -0.191 | 3.821 | -8.781 | <0.001 |

Preoperative Ca^2+^(Pre_Ca^2+^)

**(TableS2)Univariate logistic regression analysis of total thyroidectomy patients**

| Variables | Classified by the Occurrence of Hypoparathyroidism | | P | Classified by the rate of decline | | P |
| --- | --- | --- | --- | --- | --- | --- |
|  | Normal  （n=364） | Hypoparathyroidism（n=404） |  | Mild decrease  (n=576) | Significant decrease  (n=192) |  |
| Sex,*n*(%) |  |  | <0.001 |  |  | 0.078 |
| female | 272(74.7) | 342(84.7) |  | 452(78.5) | 162(84.4) |  |
| male | 92(25.3) | 62(15.3) |  | 124(21.5) | 30(15.6) |  |
| Age,*n*(%) |  |  | 0.334 |  |  | 0.477 |
| <45 | 169(46.4) | 178(44.1) |  | 256(44.4) | 91(47.4) |  |
| ≥45 | 195(53.6) | 226(55.9) |  | 320(55.6) | 101(52.6) |  |
| BMI,*n*(%) |  |  | 0.009 |  |  | 0.127 |
| <24 | 132(36.3) | 180(44.6) |  | 225(39.1) | 87(45.3) |  |
| ≥24 | 232(63.7) | 224(55.4) |  | 351(60.9) | 105(54.7) |  |
| TPOAb,*n*(%) |  |  | 0.004 |  |  | <0.001 |
| <36 | 325(89.3) | 331(81.9) |  | 510(88.5) | 149(77.6) |  |
| ≥36 | 39(10.7) | 73(18.1) |  | 66(11.5) | 43(22.4) |  |
| Erythrocyte  (10^12/L) | 4.43(4.18,4.72) | 4.38(4.13,4.63) | 0.022 | 4.39(4.16,4.68) | 4.39(4.14,4.68) | 0.495 |
| Hemoglobin(g/L) | 131 (123, 142) | 128 (119.75, 137) | 0.002 | 131(122,140) | 127(117.5,135.25) | 0.001 |
| FT3(pmol/L) | 4.23 (3.95, 4.56) | 4.2 (3.92, 4.49) | 0.196 | 4.22(3.94,4.53) | 4.18(3.93,4.51) | 0.299 |
| FT4(pmol/L) | 12.21(11.22, 13.23) | 12.2 (11.19, 13.21) | 0.992 | 12.14(11.15,13.2) | 12.36(11.44,13.34) | 0.044 |
| TSH(μIU/mL) | 1.67(1.07,2.39) | 1.7(1.16,2.44) | 0.389 | 1.69(1.16,2.43) | 1.66(1.08,2.3) | 0.372 |
| TGAb(IU/mL) | 16.5(14,36.78) | 17.1(14.5,67.8) | 0.373 | 16.6(14.2,47.72) | 17.15(14.47,69.82) | <0.001 |
| TRAb(IU/L) | 0.8(0.8, 0.8) | 0.8(0.8,0.8) | 0.656 | 0.8(0.8,0.8) | 0.8(0.8,0.8) | 0.169 |
| AST(U/L) | 20(16.75, 23) | 19(16,22) | 0.174 | 19(16,23) | 19(16,22) | 0.204 |

**Continuation Table(TableS2)**

| Variables | Classified by the Occurrence of Hypoparathyroidism | | P | Classified by the rate of decline | | P |
| --- | --- | --- | --- | --- | --- | --- |
|  | Normal  （n=364） | Hypoparathyroidism（n=404） |  | Mild decrease  (n=576) | Significant decrease  (n=192) |  |
| ALT(U/L) | 16(12, 24) | 15(11,22) | 0.091 | 15(12,23) | 15(11,22) | 0.323 |
| TBA(μmol/L) | 3.9(2.5, 6.23) | 3.95(2.8,6.2) | 0.893 | 3.9(2.5,6.23) | 4(2.88,6.12) | 0.738 |
| TP(g/L) | 69.4±4.84 | 68.93±4.76 | 0.375 | 69.25± 4.86 | 68.56± 4.59 | 0.082 |
| ALB(g/L) | 41.3(39.27, 43.23) | 40.7(38.98, 42.7) | 0.088 | 40.95(39.2,43) | 40.8(39,42.8) | 0.317 |
| TG(mmol/L) | 1.23(0.86,1.69) | 1.15(0.81,1.65) | 0.287 | 1.2(0.85,1.65) | 1.19(0.8,1.71) | 0.889 |
| TC(mmol/L) | 4.7(4.1,5.27) | 4.71(4.05,5.5) | 0.403 | 4.71(4.1,5.37) | 4.63(3.99,5.48) | 0.475 |
| HDL_C(mmol/L) | 1.28 (1.06, 1.5) | 1.31 (1.14, 1.5) | 0.287 | 1.32(1.1,1.52) | 1.25(1.11,1.44) | 0.050 |
| LDL_C(mmol/L) | 2.95(2.44,3.44) | 2.94(2.4,3.46) | 0.952 | 2.95(2.45,3.44) | 2.9(2.37,3.57) | 0.814 |
| FFA(mmol/L) | 0.37(0.26,0.51) | 0.38(0.27,0.51) | 0.523 | 0.37(0.27,0.51) | 0.39(0.29,0.52) | 0.219 |
| GLU(mmol/L) | 4.53(4.2, 4.91) | 4.52(4.16, 4.92) | 0.048 | 4.53(4.18,4.91) | 4.52(4.19,4.93) | 0.052 |
| CREA(μmol/L) | 56.15(49.27, 64.23) | 53.9(48.8, 61) | 0.041 | 55.5(49.4,63.23) | 53.45(47.68,60) | 0.094 |
| RBP(mg/L) | 33.5(29.3, 38.92) | 32.2(28.85, 38.12) | 0.045 | 33.1(29.1,38.52) | 32.05(28.9,37.92) | 0.364 |
| Pre_Ca^2+^(mmol/L) | 2.34(2.28, 2.39) | 2.33(2.27, 2.38) | 0.472 | 2.34(2.28,2.39) | 2.32(2.26,2.38) | 0.043 |
| Pre_PTH(pg/mL) | 46.04(37.06,56.8) | 43.44(34.25,55.52) | 0.022 | 42.94(34.62,52.34) | 52.08 (40.58,61.74) | <0.001 |

Preoperative Ca^2+^(Pre_Ca^2+^) ,Preoperative PTH(Pre_PTH)

**(Figure S1) ROC Curve of the Multi-Marker Combined Model**

| A.Classified by the rate of decline after total thyroidectomy |
| --- |
| 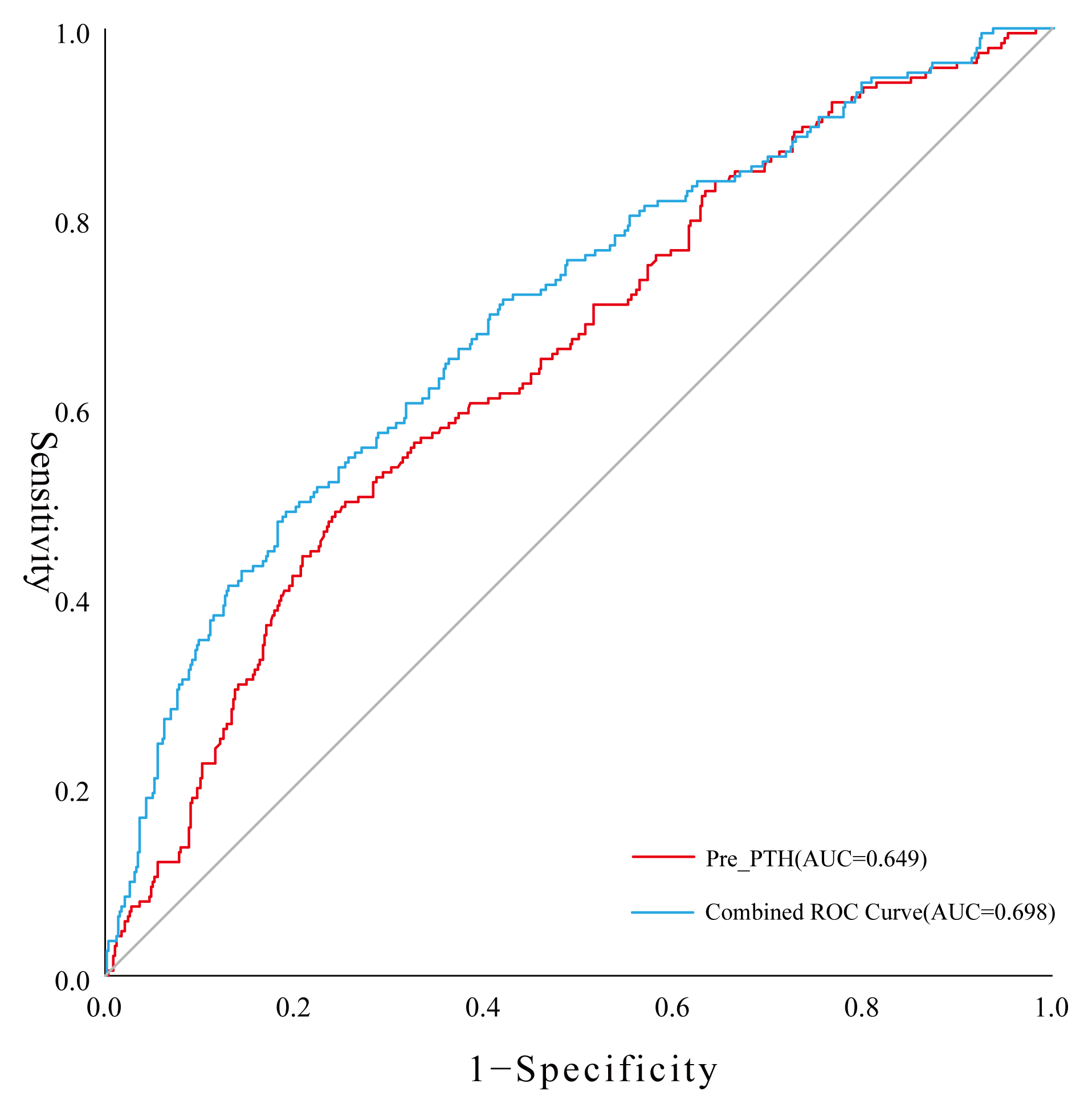 |
| B.Classified by the rate of decline after hemithyroidectomy with isthmusectomy |
| 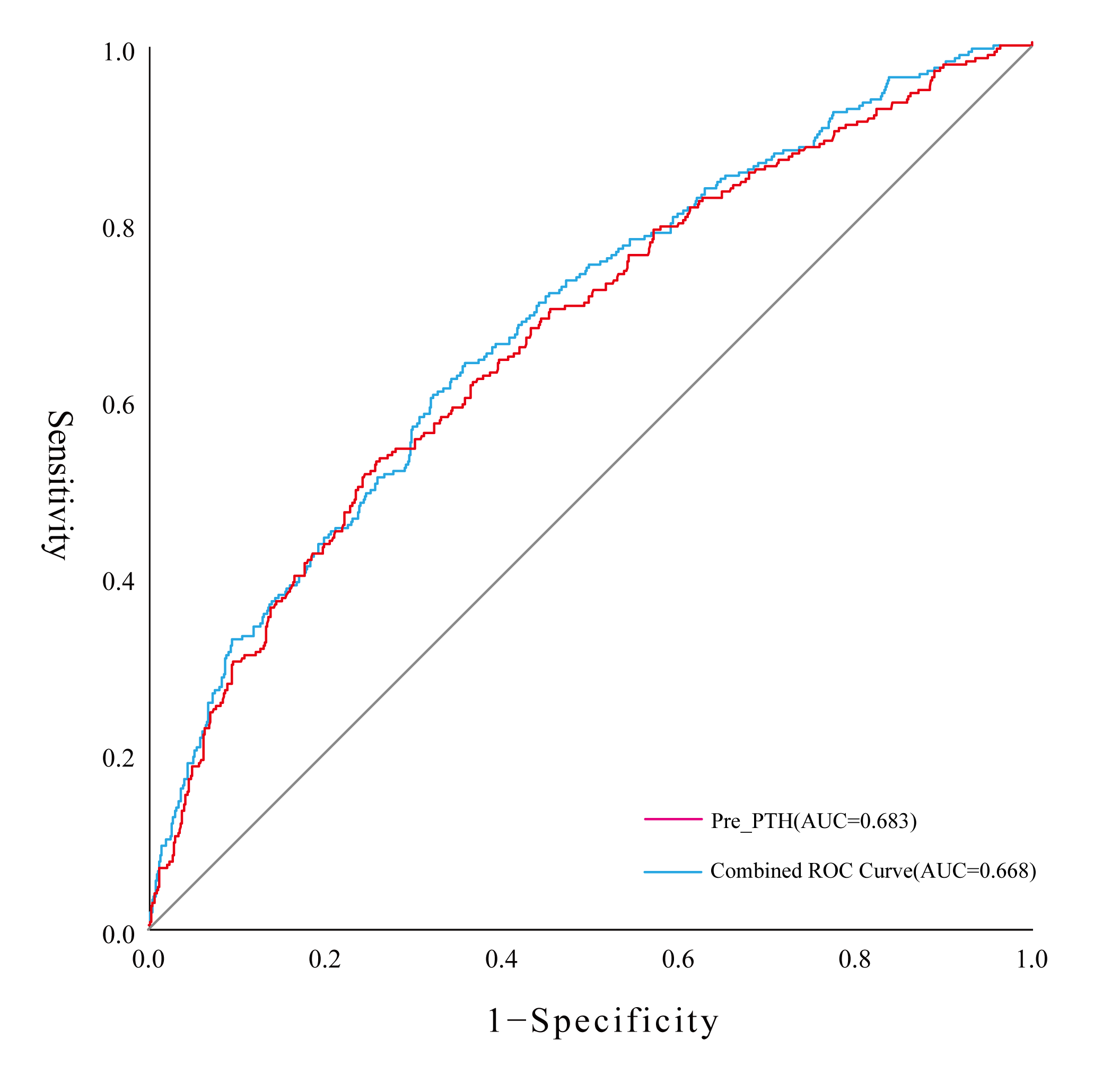 |

**(Table S3) Univariate logistic regression analysis of postoperative outcomes in hemithyroidectomy with isthmusectomy patients**

| Variables | Mild decrease  (n=777) | Significant decrease  (n=278) | p |
| --- | --- | --- | --- |
| Sex,*n*(%) |  |  | <0.001 |
| female | 577 (74) | 235 (85) |  |
| male | 200 (26) | 43 (15) |  |
| Age,*n*(%) |  |  | 0.334 |
| <45 | 393 (51) | 150 (54) |  |
| ≥45 | 384 (49) | 128 (46) |  |
| BMI,*n*(%) |  |  | 0.726 |
| <24 | 328 (42) | 114 (41) |  |
| ≥24 | 449 (58) | 164 (59) |  |
| TPOAb,*n*(%) |  |  |  |
| <36 | 667(85.8) | 238(85.6) | 0.958 |
| ≥36 | 110(14.2) | 40(14.4) |  |
| Erythrocyte  (10^12^/L) | 4.39(4.13,4.7) | 4.33(4.06,4.56) | 0.009 |
| Hemoglobin(g/L) | 130(121,141) | 128(119,136) | 0.005 |
| FT3(pmol/L) | 4.24(3.99,4.55) | 4.17(3.92,4.46) | 0.002 |
| FT4(pmol/L) | 12.24 (11.33,13.11) | 12.06(11.32,13.2) | 0.969 |
| TSH(μIU/mL) | 1.68(1.21,2.45) | 1.85(1.32,2.64) | 0.135 |
| TGAb(IU/mL) | 16.2(14.1,25.6) | 16.5(14,41.4) | 0.455 |
| TRAb(IU/L) | 0.8(0.8,0.8) | 0.8(0.8,0.8) | 0.750 |
| AST(U/L) | 19(16,22) | 18(16,21) | 0.125 |
| ALT(U/L) | 15(11,23) | 15(11,19.75) | 0.352 |
| TBA(μmol/L) | 3.9(2.5,6.4) | 3.9(2.5,6.2) | 0.830 |
| TP(g/L) | 68.4(65.4,71.8) | 68.05(65.43,71.47) | 0.950 |
| ALB(g/L) | 41.2(39.5,43) | 40.8(38.92,42.6) | 0.354 |
| TG(mmol/L) | 1.16(0.83 ,1.74) | 1.08(0.8,1.7) | 0.161 |
| TC(mmol/L) | 4.65(4.06 ,5.31) | 4.63(4.09,5.2) | 0.556 |
| HDL_C(mmol/L) | 1.29(1.09 ,1.54) | 1.29(1.14,1.47) | 0.925 |
| LDL_C(mmol/L) | 2.86(2.4,3.38) | 2.89(2.43,3.39) | 0.549 |
| FFA(mmol/L) | 0.38(0.28,0.52) | 0.41(0.29,0.54) | 0.104 |
| GLU(mmol/L) | 4.53(4.2,4.93) | 4.56(4.26,4.89) | 0.843 |
| CREA(μmol/L) | 55.7(49.6,63.6) | 54.85(49.5,61.03) | 0.079 |
| RBP(mg/L) | 33.5(29.1,38.9) | 32.75(28,37.75) | 0.056 |
| Pre_Ca^2+^(mmol/L) | 2.33(2.28,2.39) | 2.3(2.25,2.36) | <0.001 |
| Pre_PTH(pg/mL) | 41.39 (34.38,52.84) | 53.22(40.02,64.41) | <0.001 |

Preoperative Ca^2+^(Pre_Ca^2+^) ,Preoperative PTH(Pre_PTH)
